# Concurrent and predictive validity of the Alberta Infant Motor Scale and the Peabody Developmental Motor Scales-2 administered to infants born preterm in Norway

**DOI:** 10.1186/s12887-023-04402-6

**Published:** 2023-11-23

**Authors:** Tordis Ustad, Merethe Brandal, Suzann K. Campbell, Gay L. Girolami, Charlotte Sinding-Larsen, Gunn Kristin Øberg

**Affiliations:** 1 Clinic of Rehabilitation, St. Olavs Hospital, Trondheim University Hospital, St. Olavs Hospital, Postboks 3250 Torgarden, 7006 Trondheim, Norway; 2 Department of Clinical and Molecular Medicine, Faculty of Medicine and Health Sciences, Norwegian University of Science and Technology, Postboks 8900, 7491 Trondheim, Norway; 3 Department of Physical Therapy, College of Applied Health Sciences, University of Illinois at Chicago, 1301 W. Madison Street Apt.526, Chicago, IL 60612 USA; 4 Department of Physical Therapy, College of Applied Health Sciences, University of Illinois at Chicago, 1919 West Taylor Street | 455 AHSB, MC 898, Chicago, IL 60612 USA; 5 Section of Physiotherapy, Oslo University Hospital, Ullevål, Postbos 4950, Nydalen, 0424 Oslo, Norway; 6 Department of Clinical Therapeutic Services, University Hospital North Norway, Tromsø, Norway; 7 Department of Health and Care Sciences, Faculty of Health Sciences, UiT the Arctic University of Norway, Postboks 6050 Langnes, 9037 Tromsø, Norway

**Keywords:** Motor function assessment, Preterm infants, Validity study

## Abstract

**Background:**

The correlation between the Alberta Infant Motor Scale (AIMS) and the Peabody Developmental Motor Scales-2 (PDMS-2) has not previously been assessed in Norwegian infants. Our purpose was to investigate the concurrent validity of the AIMS and the PDMS-2 in a group of high-risk infants, and to investigate the predictive validity of the two tests for atypical motor function at 24 months post term age (PTA).

**Methods:**

This is a retrospective study of the AIMS and the PDMS-2 administered to infants born preterm with gestational age ≤ 32 weeks (*n* = 139) who had participated in a randomized controlled trial of early parent-administered physiotherapy. The infants’ motor development had been assessed using the AIMS and the PDMS-2 at 6- and 12-months. The primary outcome was PDMS-2 at 24-months PTA. To explore the correlation between the two tests we used Spearman’s rho. Bland Altman plots were used to detect if there were systematic differences between the measurements. Receiver-operating characteristics curves were used to calculate area under the curve as an estimate of diagnostic accuracy of the AIMS and the PDMS- with respect to motor outcome at 24 months.

**Results:**

The correlation between the AIMS and the PDMS-2 (total motor and locomotion subscale), at 6 months, was *r* = 0.44 and *r* = 0.76, and at 12 months *r* = 0.56 and* r* = 0.80 respectively. The predictive validity for atypical motor function at 24 months, assessed using the area under the curve at 6- and at 12- months, was for the AIMS 0.87 and 0.86, respectively, and for the PDMS-2 locomotion subscale 0.82 and 0.76 respectively.

**Conclusion:**

The correlation between the AIMS and the PDMS-2 locomotion subscale, at 6- and 12- months PTA, was good to excellent in a group of infants born preterm in Norway. And the AIMS and the locomotion subscale of the PDMS-2 were equally good predictors for atypical motor outcomes at 24 months PTA. These findings indicate that the AIMS and the locomotion subscale of the PDM-2, could be used interchangeable when assessing motor development in infants at 6- or 12 months of age.

**Trial registration:**

ClinicalTrials.gov NCT01089296.

**Supplementary Information:**

The online version contains supplementary material available at 10.1186/s12887-023-04402-6.

## Background

Infants born preterm are at risk of various long-term neurodevelopmental problems, a risk that increases as gestational age decreases [1]. One early marker of adverse neuro- development is the infant’s motor development [2, 3]. Several outcome measures have been developed for early identification of infants with atypical motor development. Two widely used assessment tools are the Peabody Developmental Motor Scales-2 (PDMS-2) [4] and the Alberta Infant Motor Scale (AIMS) [5], both developed to identify infants at risk for motor delay. They can also be used to evaluate change in motor development over time and can, to some extent, predict atypical motor development [6, 7]. But studies have found some cultural differences in AIMS scores, when normative data have been collected [8, 9]. The AIMS is a screening test based on observation of the infants’ spontaneous or elicited movements in four different positions [5], whereas the PDMS-2 is a comprehensive assessment of motor performance administered in a standardized manner with item scoring based on a three-point system [4].

The concurrent validity between the AIMS and the PDMS-2 has previously been examined in low birthweight infants in the United States and in high-risk infants in China [10, 11]. Concurrent validity is a type of criterion validity which refers to how the instrument correlates with another well-known instrument used in research or in clinical practice, when measured at the same time [12, 13].

As part of a multicentre randomized controlled trial of parent-administrated physiotherapy before term age, the Norwegian Physiotherapy Study in Preterm Infants (the NOPPI) [14–17], the infants were assessed using the AIMS and the PDMS-2 at 6-, 12 months post term age (PTA). The primary outcome of the NOPPI was the children’s motor development assessed by the PDMS-2 at 24 months PTA [14, 17]. Because of cultural differences in AIMS scores in previous research, it is important to investigate the concurrent validity of the AIMS compared to the PDMS-2 in Norwegian infants.

The purpose of this study was to investigate the concurrent validity of the AIMS compared to the PDMS-2 (as the well-known instrument), in a group of high-risk infants in Norway, and to investigate the predictive validity of the two tests for atypical motor development at 24 months PTA.

## Methods

### Design and study population

This is a retrospective study of the AIMS and the PDMS-2 administered to infants born preterm (with gestational age ≤ 32 weeks), who participated in the NOPPI between 2010 and 2014 [14–17]. The infants (*n* = 153) were recruited from three hospitals belonging to the National Health Service in Norway, the University Hospital of North Norway, Trondheim University Hospital and Oslo University Hospital. The sample size had been calculated based on PDMS-2 scores at 2 years PTA. A difference on gross motor and fine motor function of 0.5 standard deviation (SD) between the intervention and control group was considered clinically significant. Sixty-three infants in each group were needed to achieve a statistical power of 80% at a 0.05 (α) significance level on two-sided tests. However, only the data from 139 infants assessed using the AIMS and the PDMS-2 at 6- or 12- months PTA, and by the PDMS-2 at 24 months PTA are included in this validity study. Infants who withdrew from the study or those who did not return for more of the assessments were excluded. (Supplementary: flowchart of included infants in the NOPPI). The assessors were experienced paediatric physiotherapists, two from each hospital, who had attended a workshop, including assessment from video recordings of infants with use of the two tests.

### Assessment tools

The AIMS [5] was designed to assess gross motor development of infants from birth to walking age, and to discriminate between infants with typical and atypical motor development. The infants are assessed via observation with minimal handling or stimuli, and the assessment can be completed in 20 – 30 min. No specific toys, prompts or conditions are required while the infants are observed in four different positions: prone (21 items), supine (9 items), sitting (12 items) and standing (16 items), with handling only necessary for changing positions, or to elicit specific movements. The scores from the four positions are summed to obtain a total raw score (0 – 58) and then converted to percentiles. Scores between the 5^th^- and 15^th^ percentile indicate some motor difficulties, and below the 5^th^ percentile atypical motor development. However, studies of Brazilian infants [8] and of Flemish infants [18] have shown that there are some differences in developmental curves compared to the normative values from Canada. The normative values (n = 2200) included both preterm, fullterm and infants with congenital anomalies [5]. Canadian infants reach new motor functions earlier then Dutch and Brazilian infants. Test–retest, intra- and inter-rater reliability of the AIMS are all reported to be excellent (intraclass correlation coefficient (ICC) > 0.99) [6].

The PDMS-2 [4] is a more detailed assessment tool. It was designed to evaluate fine and gross motor skills in children from birth through 71 months, and to discriminate between typical and atypical motor development [4]. The test is administered in a standardized manner, and it takes 45 – 60 min to complete. The gross motor scale consists of 170 items organized in the following four subscales: reflexes, stationary, locomotion and object manipulation. The fine motor scale consists of two subscales, grasping and visual-motor integration. Each item is scored from 0—2 (0 = unsuccessful, 1 = clear resemblance, 2 = criterion met). The raw score of each subscale is calculated and converted to a standard score and summed to obtain a gross motor quotient, a fine motor quotient and a total motor quotient. The motor quotients have a mean of 100 and a standard deviation of 15. Motor quotients between 90 to110 indicate typical motor performance, quotients below 85 indicate difficulties in gross motor performance, and quotients below 70 indicate atypical motor performance. The normative values of the PDMS-2 are based on infants and children from 46 states in the US and from one province in Canada (*n* = 2003). The demographic characteristics reflects the status of the US population [4] Test–retest, intra- and inter-rater reliability of the PDMS-2 are reported as excellent, ICC > 0.91 [6, 7].

The concurrent validity of the AIMS and the PDMS-2 was assessed in the United States (US) and in China. Snyder et al. [10] assessed the linear correlation of 35 high-risk infants from the US, age 2 – 16 months. Pearson product-moment correlation coefficients between scores for the AIMS and PDMS-2 gross motor subscales (reflexes, stationary, and locomotion) varied from 0.84 – 0.97, with the highest correlation in the locomotion subscale. Wang et al. [11] assessed the correlation between the AIMS and the PDMS-2 in 50 high-risk infants, ages 0 – 9 months, in China. They found high degrees of correlation between the two tests. The intraclass correlation coefficients between the AIMS and the PDMS-2 subscales reflexes, stationary, and locomotion were 0.75, 0.95 and 0.97 respectively.

### Statistical analysis

Infant characteristics were calculated for perinatal and social background factors. Normality of the data was examined by the Shapiro–Wilk test. Because the data were not normally distributed at both timepoints, we used Spearman’s rho (*rs*) to explore the correlation between the AIMS and the PDMS-2. Spearman’s rho expresses the linear relationship between scores on two tests. A correlation above 0.75 is considered good to excellent, correlation between 0.50 and 0.75 is considered moderate to good, below 0.50 indicates a fair relationship and below 0.25 is considered as little or no correlation [19]. Data were analysed using the software IBM SPSS statistics version 28.

Because of the high degrees of correlation between the AIMS and the PDMS-2 found by other authors [10, 11], we also calculated the Intra Class Correlation coefficients (ICC *3,2*) to analyse the consistency between the AIMS and the PDMS-2 at 6- and 12- months. ICC values above 0.90 indicate excellent correlation, between 0.75 and 0.9 good correlation, between 0.75 and 0.50 moderate to good correlation, and below 0.50 indicates fair to no correlation [19].

Bland Altman plots were used to detect if there were systematic differences between the measurements or identify outliers. A Bland Altman plot gives a graphical presentation of the differences between two tests plotted against the average of the two tests. It visualizes the differences between two different measurements and shows the agreement with 95% confidence interval (upper and lower limit). If 95% of the scores is within these limits it shows high consistency. Furthermore, it gives a visual assessment of the scoring distribution and of potential measurement bias [20]. To construct the plot, total scores from the two tests were converted to z-scores.

Receiver-operating characteristics curves (ROC curves) were plotted to calculate the area under the curve (AUC) as an estimate of diagnostic accuracy of the AIMS and the different subscales of the PDMS-2 with respect to motor outcome at 24 months PTA. The 24-month motor outcomes were determined using the PDMS-2 total motor quotient, at either 1 standard deviation (SD) below the mean, or 2 SD below the mean of the normative sample. A perfect test has an AUC of 1.00, an AUC of 0.91 indicates excellent diagnostic accuracy, whereas 0.5 indicates discrimination no better than by chance [12, 19]. In general, the AUC should be > 0.80 to be acceptable [21]. *P*-values lower than 0.05 were considered statistically significant.

## Results

All infants were born very or extremely preterm. The infant’s characteristics and scores on the AIMS and the PMSD-2 are presented in Table 1. Fifty-one per cent of the infants were boys, 9.4% had intraventricular haemorrhage grade 1 or 2, 2.1% had intraventricular haemorrhage grade 3 or 4, and 4.3% had periventricular leukomalacia. The assessment of the infants’ motor development at 6- and 12- months showed that 24 and 18 infants respectively, received scores ≤ 5^th^ percentile by use of the AIMS, whereas 0 and 6 infants respectively, received scores ≤ 5^th^ percentile by use of the PDMS-2. At 24 months PTA only 6 infants (4.6%) had atypical motor development (< 70 on the PDMS-2) and 43 infants (33.1%) had some motor difficulties (< 85 on the PDMS-2).

**Table 1:** Clinical and demographic characteristics, and atypical motor development at 6-, 12- and 24 months PTA

**Perinatal factors** (*n* = 139)		**n**	**%**
Sex: male		71	(51.1)
Twins		31	(22.3)
Has no older siblings		88	(63.3)
Intraventricular haemorrhage grade 1–2		13	(9.4)
Intraventricular haemorrhage grade 3–4		3	(2.1)
Periventricular leukomalacia		6	(4.3)
Sepsis		16	(11.5)
Bronchopulmonary dysplasia		12	(8.6)
		**mean**	**SD**
Number of other diagnoses		1.7	(0.84)
Gestational age		29.7	(2.16)
Birth weight: grams		1383	(383)
Days of ventilation		1.70	(4.41)
Days of CPAP		16.06	(19.17)
Days with oxygen		9.69	(18.74)
**Social background factors** (*n* = 139)			
Mother’s age, years		30.99	(5.28)
Mother’s education, years		15.24 14.51	(2.77) (2.88)
Father’s education, years			
**Scores on AIMS and PDMS-2**		**n**	**%**
6 months (*n* = 137)	AIMS scores ≤ 5^th^ percentile	24	(17.5)
(*n* = 135)	PDMS-2 TMQ scores ≤ 5^th^ percentile	0	(0.0)
12 months (*n* = 129)	AIMS scores ≤ 5^th^ percentile	18	(13.9)
(*n* = 134)	PDMS-2 TMQ scores ≤ 5^th^ percentile	6	(4.5)
24 months (*n* = 130)	PDMS-2 TMQ scores ≤ 5^th^ percentile	14	(10.8)
	PDMS-2 TMQ scores < 85	43	(33.1)
	PDMS-2 TMQ scores < 70	6	(4.6)

*AIMS* Alberta Infant Motor Scale, *CPAP* Continuous positive airway pressure, *PDMS-2* Peabody Developmental Motor Scales-2, *TMQ* Total motor quotient

### Concurrent validity of the AIMS and the PDMS-2

The correlations between the AIMS and different subscales of the PDMS-2 are presented in Table 2. At both 6 and 12 months the highest correlation between the AIMS and the PDMS-2 was for the locomotion subscale. The ICC varied from 0.58 (PDMS-2 reflexes) to 0.82 (PDMS-2 locomotion) at 6 months PTA, and from 0.32 (PDMS-2 object manipulation) to 0.81 (PDMS-2 locomotion) at 12 months PTA.

**Table 2:** Correlation between the AIMS and the PDMS-2 at 6- and 12- months PTA

	**Spearman’s rho**	***p***	**ICC (*3,2*)**	**95% CI**
**PDMS-2 at 6 months**				
Total motor standard score	0.44	0.001	0.67	0.54 – 0.77
Gross motor standard score	0.50	0.001	0.66	0.52 – 0.76
**PDMS-2 Subscale at 6 months**				
Reflexes	0.48	0.001	0.58	0.41 – 0.70
Stationary	0.57	0.001	0.74	0.63 – 0.82
Locomotion	0.76	0.001	0.82	0.75 – 0.87
Grasping	0.48	0.001	0.67	0.53 – 0.76
Visual motor integration	0.47	0.001	0.63	0.48 – 0.74
**PDMS-2 Percentile at 6 months**				
Percentile total motor	0.44	0.001	0.57	0.40 – 0.70
Percentile gross motor	0.50	0.001	0.63	0.47 – 0.73
**PDMS-2 at 12 months**				
Total motor standard score	0.56	0.001	0.73	0.62 – 0.81
Gross motor standard score	0.67	0.001	0.73	0.61 – 0.80
**PDMS-2 Subscale at 12 months**				
Stationary	0.49	0.001	0.57	0.38 – 0.69
Locomotion	0.80	0.001	0.81	0.73 – 0.86
Object manipulation	0.44	0.001	0.32	0.03 – 0.52
Grasping	0.33	0.001	0.51	0.31 – 0.66
Visual motor integration	0.36	0.001	0.56	0.38 – 0.69
**PDMS-2 percentile at 12 months**				
Percentile total motor	0.57	0.001	0.70	0.58 – 0.79
Percentile gross motor	0.68	0.001	0.78	0.69 – 0.85

*CI* Confidence interval, *ICC* Intra class correlation coefficients

**Fig. 1:**
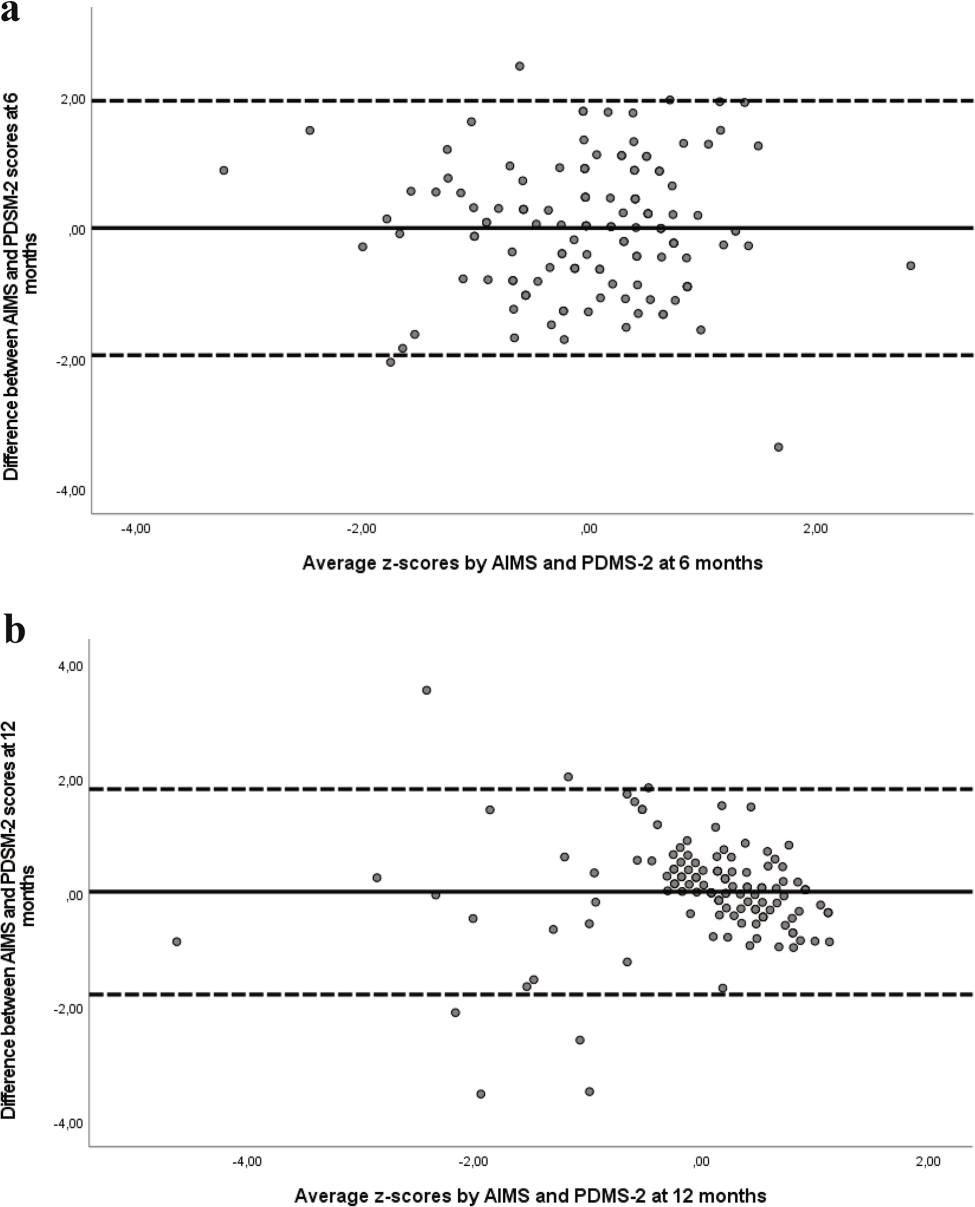
Bland–Altman plots of the difference between the AIMS and the PDMS-2 against the average of the Z-scores of the two measures. Mean difference (solid line) and ± 1.96 SD (95% of agreement) (broken lines) at 6- (**a**) and at 12- months post menstrual age (**b**)

The Bland Altman plot (Fig. 1) illustrates the differences between the AIMS and the PDMS-2 total score at 6- and 12- months. The mean differences between the two tests were close to zero which indicates very high consistency in scores on the two tests. The scores from 133 infants (98%) were within 1.96 SD of the mean difference for all observations and equally distributed above and below the zero point at 6 months, mean difference 0.0002 (SD 0.996). At 12 months the scores from 122 infants (95%) were within 1.96 SD, mean difference 0.020 (SD 0.917). Neither did we find any proportional bias between the tests at 6- or at 12 months, *t*-test 0.095 (significant level 0.96) and 0.158 (significant level 0.88), respectively.

### Predictive validity of the AIMS and the PDMS-2

**Fig. 2:**
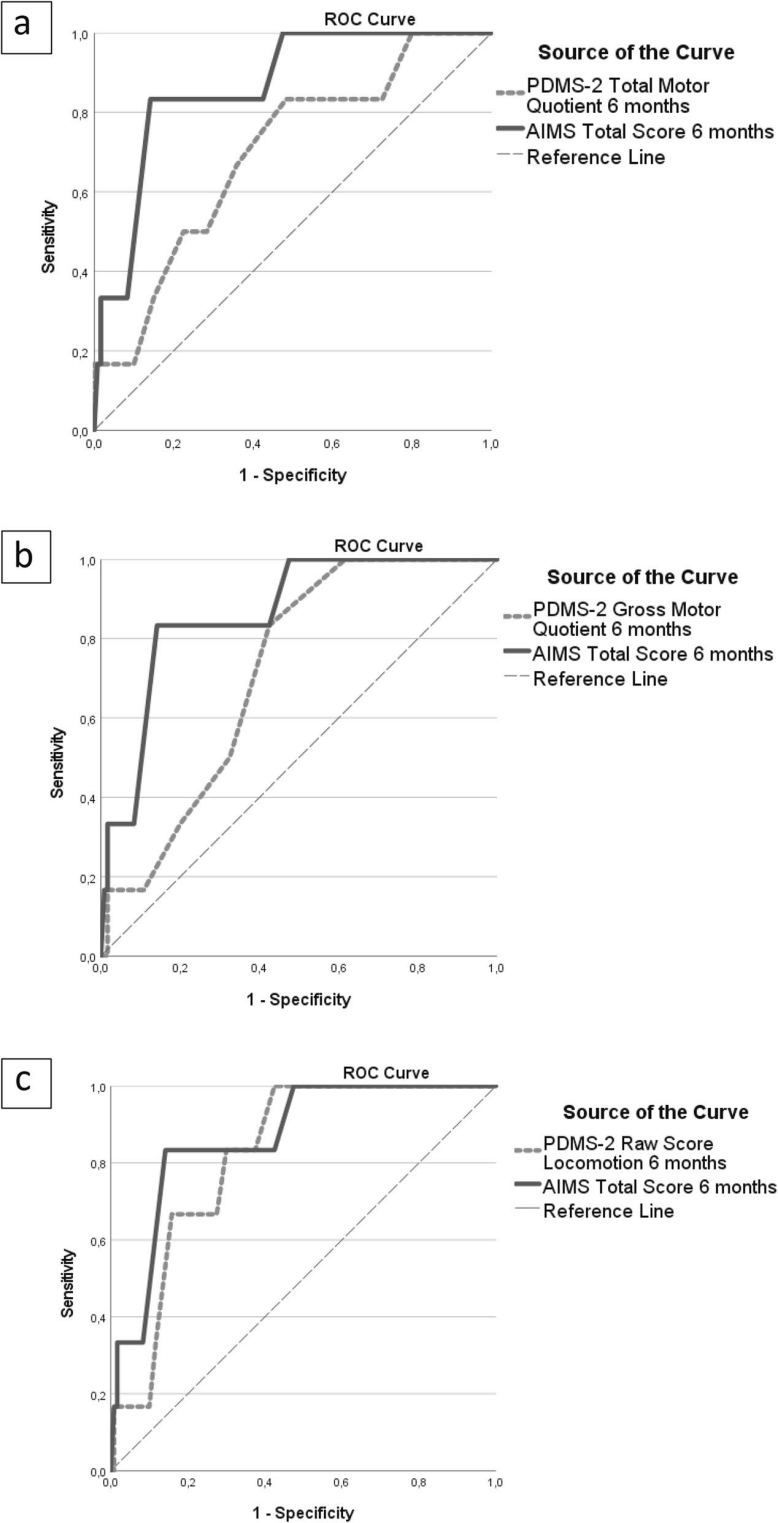
Area under the curve (AUC) at 6 months of the AIMS (solid line) and the PDMS-2 (broken line), total motor quotient (**a**), gross motor quotient (**b**) and locomotion subscale (**c**), as predictor of atypical motor development at 24 months post-term age

**Fig. 3:**
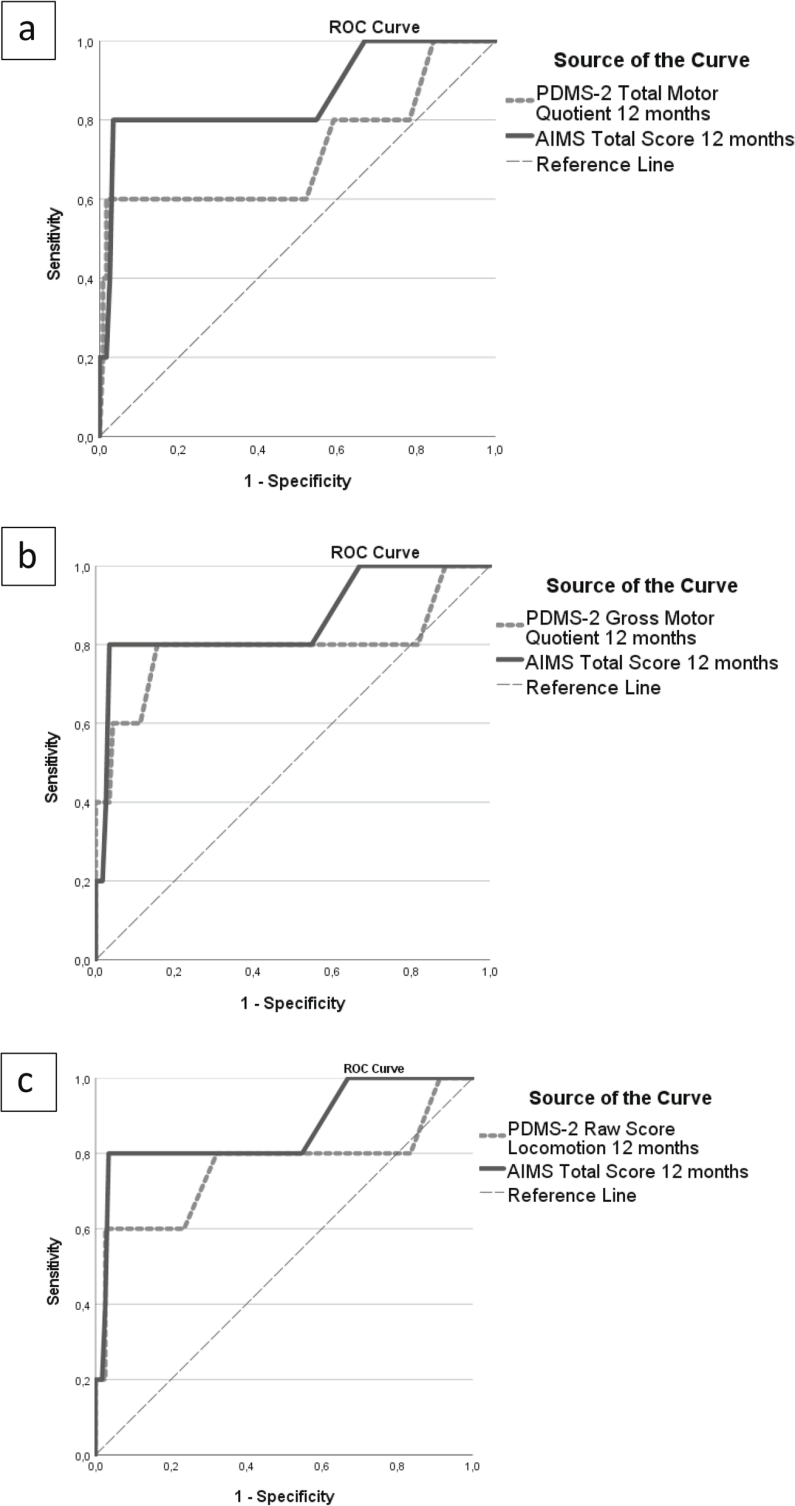
Area under the curve (AUC) at 12 months of the AIMS (solid line) and the PDMS-2 (broken line), total motor quotient (**a**), gross motor quotient (**b**) and locomotion subscale (**c**), as predictor of atypical motor development at 24 months post-term age

At 24 months PTA, 43 children showed mild delay in motor development (1 SD below the mean) and six children showed atypical motor development (2 SD below the mean) as assessed by the PDMS-2. The area under the curve (AUC) for the AIMS and different subscales of the PDMS-2 at 6- and 12- months, for cut off at 1 SD, indicated poor accuracy (Table 3). With the cut off at 2 SD, the AUC varied from 0.70 to 0.87, which indicated acceptable to excellent accuracy for both the AIMS and the PDMS-2 (total motor quotient, gross motor quotient and locomotion subscale) as shown in Figs. 2 and 3.

**Table 3:** Receiver-operating characteristics curves of the AIMS and the PDMS-2 at 6- and 12-months PTA for predicting mild delay or atypical motor development at 24 months PTA

	**Cut-off at 1 SD**		**Cut-off at 2 SD**	
	**AUC (*p*-value)**	**95% CI**	**AUC (*p*-value)**	**95% CI**
**6 months**				
AIMS score	0.64 (0.01)	0.54 – 0.74	0.87 (0.003)	0.74 – 0.99
PDMS-2 locomotion subscale	0.60 (0.06)	0.50 – 0.71	0.82 (0.008)	0.70 – 0.93
PDMS-2 gross motor quotient	0.63 (0.02)	0.52 – 0.74	0.72 (0.075)	0.57 – 0.86
PDMS-2 total motor quotient	0.62 (0.03)	0.51 – 0.73	0.70 (0.104)	0.49 – 0.90
**12 months**				
AIMS score	0.60 (0.065)	0.49 – 0.72	0.86 (0.006)	0.65 – 1.00
PDMS-2 locomotion subscale	0.69 (0.001)	0.58 – 0.80	0.76 (0.050)	0.47 – 1.00
PDMS-2 gross motor quotient	0.66 (0.003)	0.55 – 0.77	0.80 (0.007)	0.51 – 1.00
PDMS-2 total motor quotient	0.68 (0.001)	0.57 – 0.78	0.72 (0.027)	0.42 – 1.00

*AUC* Area under the curve, *CI* Confidence interval, *PTA* Post term age, *SD* Standard deviation

The AIMS at both 6- and 12 months showed the best accuracy in predicting atypical motor development at 24 months PTA, with the AUC varying from 0.87 to 0.86, respectively (Table 3). Of the six infants with atypical development at 24 months, the AIMS identified correctly five at 6 months and four at 12 months. At 12 months the AUC of the PDMS-2 (total motor quotient, gross motor quotient and locomotion subscale) varied from 0.72 to 0.80, which was lower compared to the AUC of the AIMS. Only the locomotion subscale of the PDMS-2 at 6 months, AUC 0.82, was an equally good predictor as the AIMS.

## Discussion

We found only moderate correlations between the AIMS and the PDMS-2 and its subscales in our validity study of the AIMS and the PDMS-2 in Norwegian infants born preterm (gestational age ≤ 32 weeks), except from the correlation between the AIMS and the locomotion subscale of the PDMS-2 which showed good to excellent correlation. This finding is in line with Snyder et al. [10] who also found the highest correlations between the AIMS and the locomotion subscale of the PDMS-2. As a rule, the consistency of the AIMS and the PDMS-2 was calculated to be moderate (ICC from 0.51 to 0.82). However, it was poor for the PDMS-2 object manipulation subscale at 12 months (ICC: 0.32). The different items of two tests can explain why we found the best correlation between the AIMS and the PDMS-2 locomotion subscale. The focus of the AIMS is gross motor development, specifically, how the infants move in supine, prone, sitting and standing, whereas the total score of the PDMS-2 also includes an object manipulation scale and a fine motor scale. Thus, the better correlation between the AIMS and the locomotion subscale of the PDMS-2 makes clinical sense.

In this sample of infants, more infants received a score below average on the AIMS as compared to their scores on the PDMS-2. Previous studies from Belgium [18] and from Brazil [8] have shown that infants from these two countries perform lower on the AIMS as compared to the normative values from Canada. There are no normative data from Norway, but we might speculate that Norwegian infants may also perform lower on the AIMS as compared to the Canadian norm values, which might explain our findings.

The consistency between the two tests was good as shown by the spread of the scores in the Bland Altman plot, which was evenly distributed with 98% within the limit of agreement at 6 months and within 95% at 12 months. These findings indicate that the two tests can be used interchangeably. However, there seems to be a trend at 12 months, that the difference between the methods get smaller as the average increases.

The AIMS as compared to the PDMS-2, both at 6- and at 12- months PTA, was shown to be a slightly better predictor of atypical motor development at 24 months in this group of infants born preterm. Because the AIMS is a shorter, less time-consuming test involving minimal manipulation of infants as compared to the full version of the PDMS-2, the AIMS might be the preferable tool when assessing motor development in infants born preterm at 6- and 12- months PTA. An alternative to the AIMS might be using only the gross motor scale or the locomotion subscale of the PDMS-2, when assessing the infants at 6- or at 12- months, since these were better predictors compared to the full version of the test and can also be performed within the same time as the AIMS.

### Limitations of the study

Before commencing the study, it would have been preferable to conduct an inter-rater reliability study with the involved testers, both for the AIMS and the PDMS-2. The six testers were all physiotherapists working in paediatrics, with experience using the AIMS, whereas the use of PDMS-2 was new to the physiotherapists in two of the hospitals, which might have affected the scoring.

Infants in this study were a sample of convenience (*n* = 139) of infants born preterm who participated in a multicentre early intervention study. However, the number of infants was greater than in other comparable studies [10, 11]. It is also important to note, the infants in this study received a 3-week parent-administered intervention program in the neonatal intensive care unit (NICU), beginning at postmenstrual age 34 weeks. This might be one reason very few children showed atypical motor development at 24 months PTA. Another reason might be that only infants that tolerated handling at 34 weeks, were deemed eligible to participate. Assessing motor outcomes at 24 months PTA might be too early to identify children who might have long-term motor difficulties [1].

The abovementioned limitations should be considered when generalizing the results of this study to other infants born preterm. A Rasch analysis on the AIMS showed a ceiling effect when assessing infants after the age of 9 months, because of fewer items on the test and thus lower precision for differentiating among infants [22]. Since normative values from a Norwegian population are lacking for both the AIMS and the PDMS-2, we must also be careful considering these findings.

## Conclusion

The correlation between the AIMS and the PDMS-2 locomotion subscale at 6-and 12- months PTA, was good to excellent in this group of infants born preterm (gestational age ≤ 32 weeks), in Norway. Also, the consistency between the AIMS and the locomotion subscale at both ages, and the gross motor quotient of the PDMS-2 at 12 months, was substantial. The AIMS and the locomotion subscale of the PDMS-2 were equally good predictors for atypical motor outcomes at 24 months PTA. These findings indicate that the AIMS and the locomotion subscale of the PDM-2, could be used interchangeable when assessing motor development in infants at 6- or 12 months of age.

### Supplementary Information

**Additional file 1. Supplementary.** Flow of the participants through the study.

## Data Availability

The datasets generated during and/or analysed during the current study are available from the corresponding author on reasonable request.
