# Supplementary figures and images for: Concurrent and predictive validity of the Alberta Infant Motor Scale and the Peabody Developmental Motor Scales-2 administered to infants born preterm in Norway
Source: BMC Pediatr. 2023 Nov 23;23:591. doi: 10.1186/s12887-023-04402-6 (PMC10666346; doi:10.1186/s12887-023-04402-6)

**Supplementary:** Flow of the participants through the study

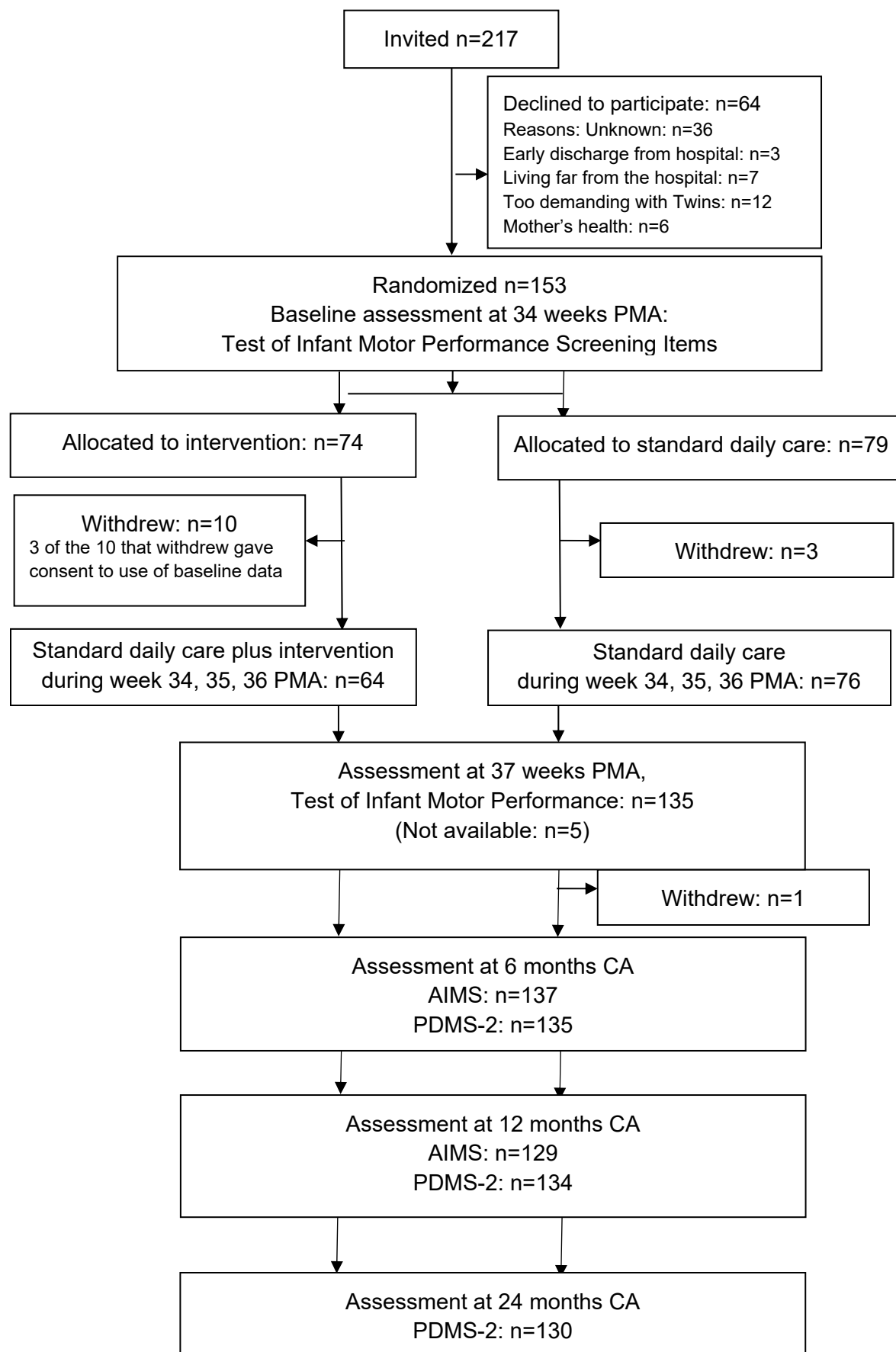

Supplement: Supplementary file 1 — Additional file 1. Supplementary. Flow of the participants through the study. [file 12887_2023_4402_MOESM1_ESM.pdf]
